# Tailoring Macromolecular Structure of Cationic Polymers towards Efficient Contact Active Antimicrobial Surfaces

**DOI:** 10.3390/polym10030241

**Published:** 2018-02-27

**Authors:** Rubén Tejero, Beatriz Gutiérrez, Daniel López, Fátima López-Fabal, José L. Gómez-Garcés, Alexandra Muñoz-Bonilla, Marta Fernández-García

**Affiliations:** 1 Instituto de Ciencia y Tecnología de Polímeros (ICTP-CSIC), C/Juan de la Cierva 3, 28006 Madrid, Spain; tejero.bonacasa@hotmail.com (R.T.); beatriz27gs@gmail.com (B.G.); daniel@ictp.csic.es (D.L.); sbonilla@ictp.csic.es (A.M.-B.); 2 Hospital Universitario de Móstoles, C/Río Júcar, s/n, Móstoles, 28935 Madrid, Spain; flopezf@salud.madrid.org (F.L.-F.); jlgarces@microb.net (J.L.G.-G.)

**Keywords:** cationic polymers, blends, surfaces, antimicrobial

## Abstract

The aim of this work is the preparation of contact active antimicrobial films by blending copolymers with quaternary ammonium salts and polyacrylonitrile as matrix material. A series of copolymers based on acrylonitrile and methacrylic monomers with quaternizable groups were designed with the purpose of investigating the influence of their chemical and structural characteristics on the antimicrobial activity of these surfaces. The biocide activity of these systems was studied against different microorganisms, such as the Gram-positive bacteria *Staphylococcus aureus* and the Gram-negative bacteria *Pseudomona aeruginosa* and the yeast *Candida parapsilosis.* The results confirmed that parameters such as flexibility and polarity of the antimicrobial polymers immobilized on the surfaces strongly affect the efficiency against microorganisms. In contrast to the behavior of copolymers in water solution, when they are tethered to the surface, the active cationic groups are less accessible and then, the mobility of the side chain is critical for a good contact with the microorganism. Blend films composed of copolymers with high positive charge density and chain mobility present up to a more than 99.999% killing efficiency against the studied microorganisms.

## 1. Introduction

Microbial adhesion and proliferation onto surfaces of medical devices or common items often leads to the spread of bacterial infections by contact, which is especially critical in hospitalized patients. According to the Centers for Disease Control and Prevention (CDC) approximately one of every 25 hospitalized patients in the U.S. develops a ‘healthcare-acquired’ infection [1], whereas one in 18 patients become infected in Europe, as stated by the European Centre for Disease Prevention and Control (ECDC) [2]. The most serious infections are surgical site infections and those associated with indwelling devices such as catheter-associated urinary tract infections. Therefore, the development of strategies to prevent or eliminate bacterial contamination on material surfaces is urgently required and has attained much interest over last years. Impregnating the surfaces with antimicrobial agents provides the potential to reduce bacterial contamination and limit the transmission of diseases. Self-disinfecting surfaces have been obtained by incorporating of a variety of antimicrobial agent including antibiotics, silver and copper compounds, light active species, alongside antimicrobial polymers [3,4]. Antimicrobial polymers offer some advantages over the rest of the existing biocides and have become increasingly important as a potential alternative. Antimicrobial polymers are in general highly active, with low potential of building up resistance and reduced toxicity [5]. Additionally, they have gained importance for the fabrication of contact active antimicrobial surfaces, which exert kill actions without releasing biocides and reducing the toxicity [6]. The antimicrobial polymers can be physically incorporated onto the surfaces and due to their high molecular weight and low diffusion coefficient, their leaching out of the surfaces is limited [7,8]. Alternatively, the polymers can be covalently anchored to the surfaces, which in most of the cases do not entail losing their biological activity [9,10]. In recent years, a variety of antimicrobial polymeric systems have been investigated including quaternary ammonium compounds, polymeric quaternary phosphonium salts, guanidine containing polymers and halogen polymers (i.e., *N*-halamines) among others [11,12]. Most of the studied systems are polycations, in particular those with quaternary nitrogen atoms [13,14]. Although their mechanism of action is not fully understood, these polycationic structures interact electrostatically with the negative charged bacterial membrane, causing disruption of the wall and the posterior death of the microorganism. Many investigations have been focused on discussing the optimal chemical structure and the factors affecting the antimicrobial activity of these cationic polymers such as the hydrophobic/hydrophilic balance, the charge density, the length of the alkyl chain as well as molecular weight of the polymers [15,16]. However, most of these studies imply analyses in solution rather than on surfaces. When the polymer is attached onto a surfaces its mobility can be reduced and the accessibility of the active groups limited.

Herein, we systematically study the influence of polymer chemical structure on the antimicrobial activity of polymeric films. For this purpose, we prepared films of blends, consisting of a series of cationic copolymers physically blended with polyacrylonitrile (PAN). Polyacrylonitrile was selected as model material with excellent properties such as thermal and UV stability, chemical resistance, high strength and modulus of elasticity, that make it a desired material for a variety of biomedical uses, such as protein filtration and hemodialysis membranes [17]. As antimicrobial copolymer incorporated to PAN, we employ a series of methacrylic copolymers bearing two cationic groups per monomeric units previously designed and synthesized by our group [18]. These structures are based on monomeric units with 1,3-thiazolium and 1,2,3-triazolium side-chain groups (MTA#), which demonstrated broad spectrum of antimicrobial activity in solution against Gram-negative and Gram-positive bacteria and fungi. In this series of copolymers, it was varied several structural and chemical parameters with the aim of investigating their influence on the antimicrobial activity when there are on a surface. In particular, these copolymers were obtained by copolymerization of MTA# units and acrylonitrile monomer, varying the final chemical composition, that is, the hydrophobic/hydrophilic balance. In addition, several MTA# monomers were employed in the copolymerization, in which positive charge density, the length of the side chain, its flexibility and polarity were also varied.

## 2. Experimental Part

### 2.1. Materials

**Figure 1:**
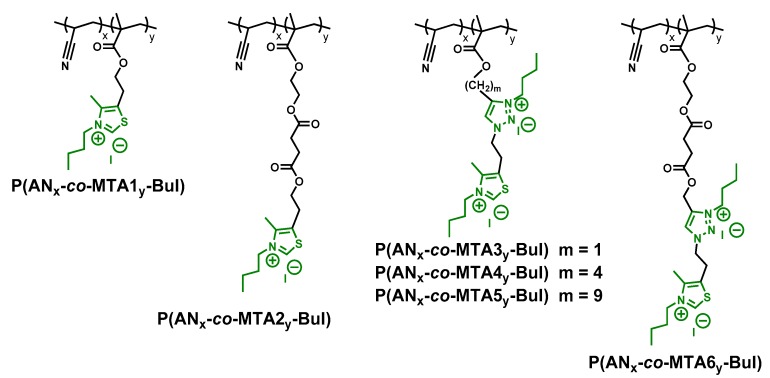
Quaternized copolymers of acrylonitrile and MTA# monomers.

Several P(AN_x_-*co*-MTA#_y_-Bu) statistical copolymers quaternized with butyl iodide were synthesized as previously reported by our group (see Figure 1) [18,19]. Polyacrylonitrile (PAN, *M_n_* = 150 kDa) was supplied by Sigma Aldrich (Saint-Quentin-Fallavier, France) and was used without previous purification. *N*,*N*-dimethylformamide (DMF, 99.8%) provided by Alfa-Aesar (Karlsrue, Germany) and ethanol (EtOH, 99.9%) from Scharlau Chemie (Munich, Germany) were used as received. Sodium chloride (NaCl, 0.9%, BioXtra, suitable for cell cultures, Saint-Quentin-Fallavier, France), saline phosphate buffered saline (PBS, pH 7.4) and formalin (10%, neutral buffer) were purchased from Aldrich (Saint-Quentin-Fallavier, France) and used directly.

For the microbiological assays: Sheep blood (5%) Columbia Agar plates were purchased from bioMérieux (Madrid, Spain) and BBLTM Mueller Hinton broth was purchased from Becton, Dickinson and Company (Madrid, Spain) and was used as a microbial growth media. American Type Culture Collection (ATCC): Gram-negative *Pseudomonas aeruginosa* (*P. aeruginosa*, ATCC 27853) and Gram-positive *Staphylococcus aureus* (*S. aureus*, ATCC 29213) bacteria and *Candida parapsilosis* (*C. parapsilosis*, ATCC 22019) yeast were obtained from Oxoid^TM^ (Madrid, Spain). Microorganisms were incubated for 24 h for bacteria and 48 h for yeast at 37 °C in a Jouan IQ050 (Winchester, VA, USA) incubator. The optical density of the microorganism suspensions was measured in McFarland units proportional to microorganism concentration by a DensiCHEK™ Plus (VITEK, bioMérieux, Madrid, Spain).

### 2.2. Antimicrobial Films Preparation and Characterization

The films were prepared by casting process from polymer blend solutions in DMF at a final polymer concentration of 10 wt %. These solutions were prepared by dissolving PAN and the corresponding quaternized copolymer P(AN_x_-*co*-MTA#_y_-Bu) in a PAN/copolymer ratio of 70/30 by weight. Each solution was filtered through a fiberglass filter (Symta, Madrid, Spain) with a pore diameter of 3.1 μm and then, spread in a flat Petri dish. The solvent was first eliminated at room temperature, followed by a heating treatment at 50 °C for 18 h in an oven and finally dried under vacuum until constant weight. 

The obtained films were characterized by contact angle measurements performed in a KSV Theta goniometer. The volume of the droplets was controlled to be 3.0 μL and the images of the water droplets were capture with a charge coupled device camera for the determination of the contact angle values. The morphology of the surfaces was analyzed by scanning electron microscopy (SEM) in Philips XL30 microscope (Eindhoven, The Netherlands) with an acceleration voltage of 25 kV. The samples were pre-coated with gold-palladium (80/20).

### 2.3. Evaluation of Antimicrobial Activity of the Films

Antimicrobial activity of blend films was determined following the E2149-01 standard method of the American Society for Testing and Materials (ASTM) [20] against *P. aeruginosa*, *S. aureus* and *C. parapsilosis*. Initially, the microbial strains were grown on 5% sheep blood Columbia agar plates, dispersed, and adjusted to a turbidity equivalent to 0.5 McFarland standards (~10^8^ colony-forming units per mL, CFU/mL) with sterile saline solution. Subsequently, the working bacterial suspension (~5 × 10^5^ CFU/mL) was obtained by 200-fold dilution with phosphate buffered saline. Films were previously sterilized by washing with ethanol and exposure to UV radiation during 30 min. Then, each sample was introduced in a sterile falcon tube and 10 mL of inoculum with ~5 × 10^5^ CFU/mL were added. Falcon tubes with only the inoculum and PAN film without the copolymers were also prepared as control experiments. All samples were shaken at ambient temperature at 150 rpm for 24 h. Bacterial concentrations at time 0 and after 24 h were calculated by the plate count method performing 1:10 serial dilutions, followed by the drop plate technique. Three films of each sample were evaluated and plated them by duplicate. The percentage reduction was estimated from the average of the results.

### 2.4. Characterization of Microorganisms after Exposure to the Antimicrobial Surface by SEM 

Microbial strains (*P. aeruginosa*, *S. aureus* and *C. parapsilosis*) were cultured in a similar protocol as antimicrobial measurements. The microorganism suspensions, ~5 × 10^5^ CFU mL^−1^, were treated with the films blend and incubated for 24 h (48 h for *C. parapsilosis*). After incubation, the microbes were fixed on amorphous carbon-coated copper grids with 10% formalin solution for 60 min at room temperature. Subsequently, the grids were washed twice with PBS and water, and finally dried for 10 min with ethanol/water mixtures increasing sequentially the ethanol content from 30 to 50, 70 and 100%. The dehydrated samples were dried at room temperature, and imaged with field emission scanning electron microscopy (FE-SEM) Hitachi SU 8000 from Hitachi High-Technologies (Tokyo, Japan) at 30 kV. 

The adherent microorganisms (*S. aureus* bacteria) were also visualized by FE-SEM. Films were incubated with bacterial suspension (~5 × 10^5^ CFU mL^−1^) for 2 h, then the surfaces were carefully rinsed several times with PBS, and the bacteria were fixed using the previous protocol described. The micrographs of the films were recorded by FE-SEM with a Hitachi SU 8000 at 30 kV.

## 3. Results and Discussion

### 3.1. Antimicrobial Copolymer Design and Film Preparation

Several statistical copolymer systems were investigated as antimicrobial agents to modify polymeric surfaces (Figure 1). The copolymers consist of acrylonitrile (AN) units statistically copolymerized until complete conversion by radical copolymerization with different methacrylic monomers containing 1,3-thiazole pendant groups, MTA#, in which the final copolymer composition was varied, with mole fractions of AN in the copolymer, f_AN_: 0.2, 0.4, 0.6 and 0.8. The copolymers presented molecular weights between 21,000 to 135,000 g/mol as determined by size exclusion chromatography (SEC) [18].

Several antimicrobial monomer structures (MTA#) were designed and selected in order to study the influence of different structural parameters on the antimicrobial activity, on one hand the length and flexibility of the side chain, secondly its polarity and, on the three hand, the incorporation of additional antimicrobial functionality in further quaternization reaction, 1,3-thiazole and 1,2,3-triazole moieties. The positive charge and the hydrophobic/hydrophilic balance are fundamental aspects affecting the antimicrobial activity of polymers in solution. In the particular case of antimicrobial films, the behavior of the antimicrobial polymer might vary significantly as the chains are attached onto the surface and the mobility might be partially impeded. Then, in addition to the positive charge, the structure of the side chain, in terms of polarity and mobility, is expected to be crucial. Regarding the positive charge, the MTA1 and MTA2 units only bear thiazole functionality whereas MTA3, MTA4, MTA5 and MTA6 contain also a triazole group, thus two groups per monomer susceptible of quaternization. Also, it was studied the influence of the MTA# comonomer composition on the antimicrobial activity, that is the hydrophilic/hydrophobic balances. Thereby, several compositions were prepared for each system, varying the f_AN_. As above mentioned, in addition to the charge density and the hydrophobic/hydrophilic balance, the flexibility of the side chain, the length of the side chain was varied; MTA2 with a long side chain of ethyl succinate, showed higher flexibility than MTA1 leading polymers with lower glass transition temperature (*T_g_*) [21]. The flexibility of the side chain was also varied in polymers bearing both functional groups, MTA3, MTA4, MTA5 and MTA6 in which the length of the lateral chain was also systematically changed. In the copolymers MTA3, MTA4 and MTA5 the side chain incorporates alkyl group, methyl, butyl and nonyl, respectively; whereas the MTA6 contains a succinate group, which is a more polar group. Finally, all the copolymers were quaternized with butyl iodide to obtain the polycationic and antimicrobial systems. Table 1 summarizes the *T_g_* of all the copolymers studied in this work. 

From all these statistical copolymers, blend films were prepared by a casting process by dissolving PAN and the corresponding quaternized copolymer P(AN_x_-*co*-MTA#_y_-Bu) in DMF at a final concentration of 10 wt %. The ratio between PAN and copolymers was fixed to 70/30 by weight. Once the solvent was evaporated at room temperature, the films were further dried at 50 °C and the complete solvent removal was checked on each film by ATR-FTIR spectroscopy. 

**Figure 2:**
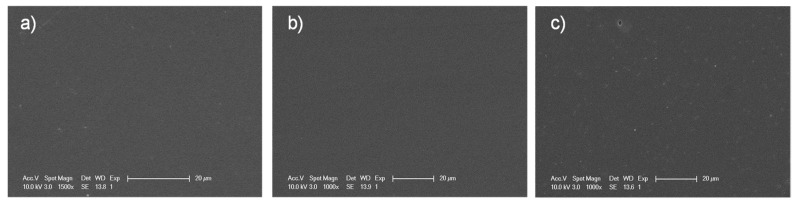
SEM images of the films containing the copolymers: (**a**) P(AN_0.6_-*co*-MTA1_0.4_-Bu), (**b**) P(AN_0.6_-*co*-MTA4_0.4_-Bu) and (**c**) P(AN_0.6_-*co*-MTA6_0.4_-Bu).

All the obtained films were optically transparent and their homogeneity were further analyzed by SEM. Figure 2 shows as examples, the SEM images of films containing P(AN_0.6_-*co*-MTA1_0.4_-Bu), P(AN_0.6_-*co*-MTA4_0.4_-Bu) and P(AN_0.6_-*co*-MTA6_0.4_-Bu) copolymers. It is clearly observed that the surfaces are homogeneous and smooth with only few irregularities such as some pores, which demonstrated that the incorporation of the copolymers did not alter the topography of the PAN.

The surface wettability can give information of the surface functionality of the obtained films. Water contact angle values of the films are also collected in Table 1, while the contact angle for the PAN film was found to be 79 ± 3, close to those reported in the literature [22]. The θ values of the blends containing the copolymers slightly decrease as a result of the incorporation of the cationic copolymers. Remarkably, higher contact angles are found for the blends containing the copolymers P(AN_x_-*co*-MTA4_y_-Bu) and P(AN_x_-*co*-MTA5_y_-Bu), which is expected as these copolymers bear long alkyl side chains, butyl and nonyl, respectively. When chemical composition of the copolymer is varied for each series, only small differences were appreciated, with a reduction of the contact angle as the content of MTA# diminished in the copolymers, that is higher f_AN_ values. 

### 3.2. Evaluation of the Antimicrobial Activity of the Blend Films

**Figure 3:**
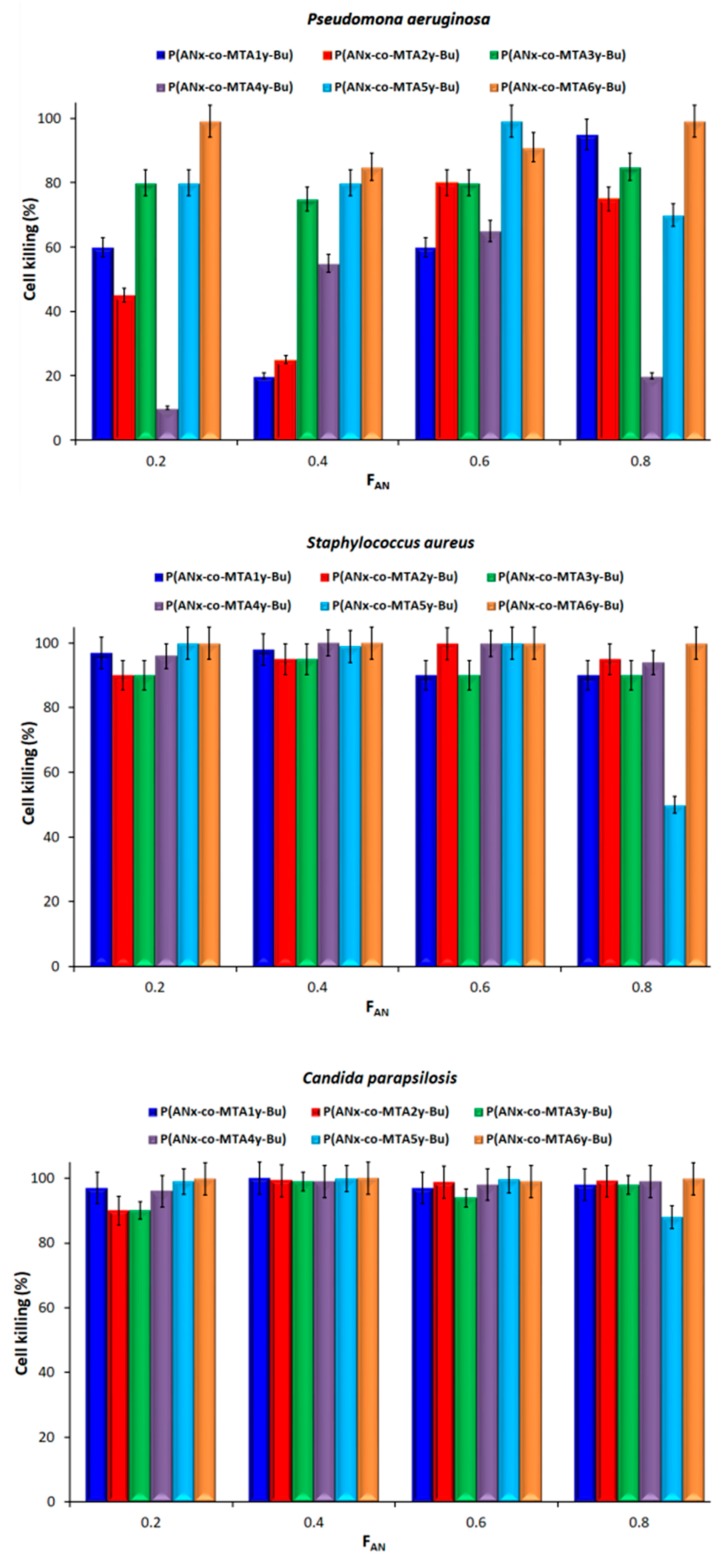
Cell-killing percentage of the contact active films for *P. aeruginosa*, *S. aureus and C. parapsilosis* microorganisms.

Next, the antimicrobial activity of the prepared films containing the different series of antimicrobial copolymers was tested against Gram-negative *P. aeruginosa*, Gram-positive *S. aureus* bacteria and *C. parapsilosis* yeast after 24 and 48 h of incubation, respectively. It has to be mentioned that the copolymers were not directly water soluble, and then any possible leaching out of the film is avoided. Figure 3 summarizes the cell-killing percentage for each microorganisms expressed with respect to control experiments in which the bacterial reduction was null (experiments done on films prepared from exclusively PAN, and without any films). 

It is worthy to remark that the films containing the P(AN_x_-*co*-MTA#_y_-Bu) copolymers practically did not present activity against Gram-negative bacteria. Previous investigations of these copolymers in aqueous-DMSO media reveal, in general, high activity against both Gram-positive and Gram-negative bacteria and against *C. parapsilosis*, showing broad spectrum activity [18]. In contrast, when the copolymers are immobilized on the films the activity against Gram-negative bacteria is significantly reduced, meaning that a stronger and closer interaction between the polymer chain and the bacterial membrane is necessary to kill Gram-negative bacteria with a double membrane. Therefore, it seems that the mobility of the copolymers is crucial for the bactericidal behavior of these series of copolymers. However, the films show acceptable activity against Gram-positive *S. aureus* bacterium and *C. parapsilosis* yeast, with the exception of samples containing P(AN_x_-*co*-MTA1_y_-Bu), P(AN_x_-*co*-MTA2_y_-Bu) and P(AN_x_-*co*-MTA3_y_-Bu) copolymers that exhibit low activity against *S. aureus* bacteria. The P(AN_x_-*co*-MTA1_y_-Bu) and P(AN_x_-*co*-MTA2_y_-Bu) copolymers contain monomeric units with only thiazolium moieties, thereby with lower cationic charge density in comparison with the rest of the structures that contains an addition triazolium group. Therefore, in principle, an apparently high charge density is required to achieve good antibacterial activity in such polymer films with relatively low content of antimicrobial copolymer related to the PAN homopolymer, 30/70. However, the copolymer P(AN_x_-*co*-MTA3_y_-Bu) bears thiazolium and triazolium groups in its structures, thus high charge density but still showed low activity. 

Contrary, in solution all these copolymers demonstrated considerable efficacy against all the microorganisms, suggesting that when the copolymer is embed in the films the active thiazolium and/or triazolium groups might be less accessible. Then, although the charge density should be important, the cationic groups have to be available to contact with the microorganisms. Therefore, the flexibility and the polarity of the side chain are expected to be decisive parameters in the antimicrobial activity. In effect, as the length of the alkyl chain augments, with butyl and nonyl as alkyl group spacer, for the P(AN_x_-*co*-MTA4_y_-Bu) and P(AN_x_-*co*-MTA5_y_-Bu) copolymers, respectively, the cell-killing percentage remarkably increases, up to more than 99.999% killing efficiency in some cases. However, no significant differences can be observed when comparing both systems, with butyl and nonyl. This can be explained by the fact that as the alkyl group is longer and more flexible, the hydrophobicity of the chains is also higher, which can provoke the constriction of the chain and disfavor its accessibility. Indeed, the copolymer P(AN_x_-*co*-MTA6_y_-Bu) with a long side chain of succinate, a more polar group, exhibits excellent antimicrobial activity against *S. aureus* bacteria and *C. parapsilosis* yeast for all the copolymer compositions, and relatively acceptable against Gram-negative *P. aeruginosa* bacteria. This copolymer may present a good balance between flexibility and polarity, thus their active cationic groups are available for killing contact. 

The composition of the copolymer is another parameter that might influence the antimicrobial activity of the copolymer. In principle, copolymer with large content of antimicrobial copolymer, low f_AN_, would exhibit better antimicrobial activity as the content of cationic group is higher and their *T_g_* lower. However, it can be observed from the data that intermediate compositions showed better performances, which demonstrates that the hydrophobic/hydrophilic balance of the copolymer is also important in the interaction with the bacterial membrane as previously reported in many studies [15,23,24]. 

In summary, for antimicrobial polymers immobilized on surfaces the flexibility and polarity of the side chain are crucial parameters for enhancing the accessibility of the active cationic groups. In addition, an intermediate copolymer composition with adequate hydrophobic/hydrophilic balance is desired for a good antimicrobial activity of the copolymer attached onto surfaces. 

### 3.3. Evaluation of the Morphological Changes of Microbes

**Figure 4:**
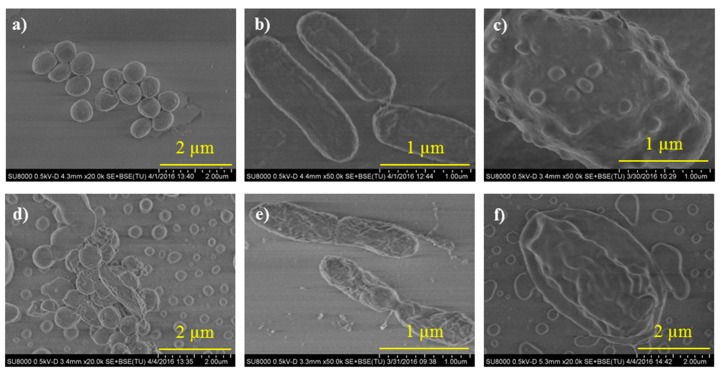
FE-SEM images of: (**a**,**d**) *S. aureus*; (**b**,**e**) *P. aeruginosa*; and (**c**,**f**) *C. parapsilosis* after incubation for 24 h (48 h for *C. parapsilosis*) on control films of PAN (**a**–**c**) and on films of the blend PAN/P(AN_0.6_-*co*-MTA4_0.4_-Bu) (**d**–**f**).

The morphological changes of microorganisms, *S. aureus*, *P. aeruginosa* and *C. parapsilosis*, after incubation with the antimicrobial films during 24 h (48 h for *C. parapsilosis*) were observed by Field Emission Scanning Electron Microscopy (FE-SEM) as shown in Figure 4. In particular, the micrographs show the results with the films containing the copolymer P(AN_0.6_-*co*-MTA4_0.4_-Bu). Control experiments were also carried out, in which the microbes were incubated onto films obtained from exclusively PAN solutions. It is clearly observed that the morphology of the cells incubated with the antimicrobial films significantly changed with respect to the cells incubated onto control PAN films as observed in other studies [25]. The shape of all studied microorganisms becomes more expanded and irregular. The microbes tend to be stuck together and in some cases, especially in Gram-positive *S. aureus* bacteria (Figure 4d), membrane-fusion events took place. The cell membranes of the *C. parapsilosis* yeast (Figure 4f) also present damaged structure in which the intracellular material leaked out from the cells. In the case of the Gram-negative *P. aeruginosa* bacteria, although the damage seems less accused, the bacterial cell membrane was also distorted (Figure 4e). 

Therefore, these results demonstrated that the mechanism of action of these antimicrobial surfaces is by cell membrane disrupting due to hydrophobic interactions and electrostatic interactions between the negatively charged cell walls and the positively charged copolymers [26,27,28,29].

**Figure 5:**
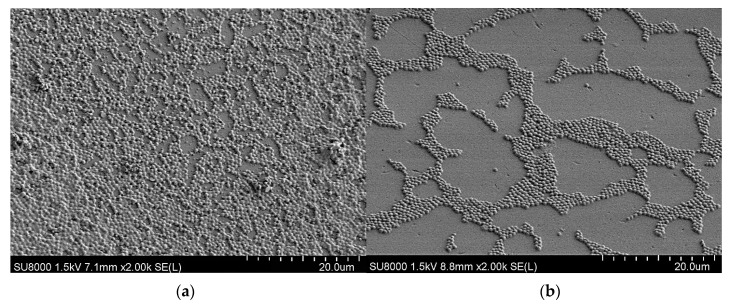
FE-SEM images of *S. aureus* on PAN films (**a**) in the absence and (**b**) in the presence of the antimicrobial copolymer P(AN_0.6_-*co*-MTA4_0.4_-Bu) after 2 h of contact.

Figure 5 shows low magnification FE-SEM micrographs of the film surfaces obtained from PAN and from the blend of PAN and P(AN_0.6_-*co*-MTA4_0.4_-Bu) after incubation with *S. aureus* bacteria during 2 h. It is clearly observed a significant reduction in the bacterial population in comparison with the control film, made from PAN without antimicrobial copolymer.

## 4. Conclusions

In this work, contact active antimicrobial films were prepared by simple blending process incorporating cationic copolymers into polyacrylonitrile matrix, a polymeric material extensively used in biomedical applications. A series of cationic copolymers with high charge density was selected to investigate systematically the influence of the macromolecular structure on the biocidal efficiency, when the cationic polymers are tethered on a surface rather than in solution. The results showed that the films presented low activity against Gram-negative *P. aeruginosa* bacteria, in contrast to the data obtained in solution in which the cationic polymers were highly efficient. Besides, it was demonstrated that the macromolecular structure of the cationic polymers strongly affects the biocidal activity of the blend films, in addition to the influence of chemical composition and positive charge density. Remarkably, films composed of copolymers with high chain mobility exhibit better cell killing efficiency, more than 99.999%. Thus, we can conclude that when cationic polymers are enclosed on surface, parameters such as the length, flexibility and polarity of the side chain are crucial to enhance the accessibility of the active groups for killing the microorganisms by surface contact. 

## Figures and Tables

**Table 1:** Chemical composition of the antimicrobial copolymers used in the blends with PAN, their glass transition temperatures (*T_g_*), and the static water contact angles (θ).

Copolymer in the Blend	f_AN_	*T_g_* (°C) ^a^	θ (°)
P(AN_x_-*co*-MTA1_y_-Bu)	0.2	48	69 ± 3
	0.4	52	67 ± 2
	0.6	60	67 ± 2
	0.8	69	65 ± 1
P(AN_x_-*co*-MTA2_y_-Bu)	0.2	10	66 ± 3
	0.4	12	64 ± 3
	0.6	22	65 ± 2
	0.8	32	61 ± 2
P(AN_x_-*co*-MTA3_y_-Bu)	0.2	61	64 ± 2
	0.4	63	63 ± 2
	0.6	71	70 ± 4
	0.8	74	62 ± 3
P(AN_x_-*co*-MTA4_y_-Bu)	0.2	25	79 ± 3
	0.4	29	75 ± 3
	0.6	44	72 ± 3
	0.8	55	70 ± 3
P(AN_x_-*co*-MTA5_y_-Bu)	0.2	-7	73 ± 2
	0.4	0	70 ± 3
	0.6	8	78 ± 3
	0.8	25	75 ± 3
P(AN_x_-*co*-MTA6_y_-Bu)	0.2	20	60 ± 2
	0.4	23	69 ± 3
	0.6	32	65 ± 2
	0.8	45	65 ± 2

^a^ Data obtained from reference [21].
